# GWAS analysis of a depression cohort defined by an EHR-phenotyping algorithm reveals the role of immune regulations in depression risk

**DOI:** 10.3389/fgene.2026.1818653

**Published:** 2026-06-17

**Authors:** Su Xian, David Carrell, Jordan W. Smoller, Wei-Qi Wei, Gail P. Jarvik, David R. Crosslin

**Affiliations:** 1 Department of Biomedical Informatics and Medical Education, University of Washington, Seattle, WA, United States; 2 Kaiser Permanente Washington Health Research Institute, Seattle, WA, United States; 3 Psychiatric and Neurodevelopmental Genetics Unit, Massachusetts General Hospital, Center for Genomic Medicine, Boston, MA, United States; 4 Department of Psychiatry, Massachusetts General Hospital, Center for Precision Psychiatry, Boston, MA, United States; 5 Department of Biomedical Informatics, Vanderbilt University Medical Center, Nashville, TN, United States; 6 Division of Medical Genetics, Department of Medicine, University of Washington, Seattle, WA, United States; 7 Department of Genome Sciences, University of Washington, Seattle, WA, United States; 8 Division of Biomedical Informatics and Genomics, Department of Medicine, Tulane University, New Orleans, LA, United States

**Keywords:** depression, EHR (electronic health record), genetics, GWAS, immune regulation, phenotyping algorithms

## Abstract

**Introduction:**

Depression is a common psychiatric disorder and a leading cause of disability. Large-scale genomic studies have identified common variants associated with depression. However, researchers often rely on self-reported phenotypes, domain expertise-defined rules, and simple diagnostic codes (ICD9, ICD10) to identify depression participants, which suffers from inconsistent cohort definition and limited sample sizes. Thus, there is a lack of validated, efficient EHR phenotyping algorithms that precisely recognize depression cases.

**Methods:**

We implemented a validated EHR phenotyping algorithm to construct a cohort of individuals with depression (11,532 cases and 39,631 controls, total n = 51,163) and conducted a genome-wide association study (GWAS) using this cohort. We validated the EHR-derived depression cohort using LDSC regression, comparing genetic similarities between our cohort and existing large meta-analyses. Top-ranked SNPs were selected and annotated to investigate downstream biological pathways and potential mechanisms that interfere with depression susceptibility.

**Results:**

Our study reproduced previously identified genetic associations (*PHF5A, KCNG2*) with depression susceptibility. We also identified novel SNPs within the HLA region and IGVH region, suggesting an association between immune function and depression phenotype. We also demonstrated the robustness of the phenotyping algorithm through genetic correlation analysis (LDSC), showing a highly genetic similarity (rg = 0.8317, *P* = 1.7758e-11) between our cohort and large meta-analysis cohorts of major depressive disorder.

**Conclusion:**

Our results demonstrate a robust validation of the EHR-based depression phenotyping algorithm using genetic analysis while providing novel genetic associations between depression and immune functions.

## Introduction

Depression is a pervasive mental health disorder characterized by persistent feelings of sadness, loss of interest, and a range of cognitive and physical symptoms. It affects millions of individuals worldwide, causing significant personal suffering, impaired functioning, and socioeconomic burden (Wang et al., 2017). The etiology of depression is complex, involving the interplay of genetic, environmental, and psychological factors (Penninx et al., 2013; Sullivan et al., 2000). While environmental stressors and psychosocial factors have been extensively studied, genetic factors have emerged as key contributors to the susceptibility and development of depression (Wray et al., 2018).

Understanding the genetic basis of depression is of paramount importance for several reasons. Firstly, it provides crucial insights into the underlying biological mechanisms, potentially leading to the development of more targeted and effective therapeutic interventions. Secondly, genetic studies can aid in the identification of individuals at higher risk of developing depression, facilitating early detection and intervention.

Genetic models, such GWAS, have identified a large number of genomic regions associated with depression susceptibility, implicating various biological pathways, including neurodevelopment, neurotransmission, and immune response (Wei et al., 2016; Howard et al., 2019). Nonetheless, the effect sizes of the identified variants have been modest, indicating that depression is highly likely polygenic, with each genetic variant conferring a small effect size.

Most GWAS studies used diagnosis codes or expertise-based manual effort to build the cohort, lacking a unified phenotyping method that can precisely identify depression patients in a clinical setup (Levey et al., 2021; Levey et al., 2020). Recently, some meta-analyses included multiple datasets, boosting the sample size and facilitating the identification of novel genetic associations (Wray et al., 2018; Levey et al., 2021; Als et al., 2023) Researchers also explored some EHR-based phenotyping algorithms but detected certain levels of inconsistency (Cai et al., 2020). In this study, we aim to contribute to the growing body of knowledge on the genetic basis of depression by conducting a GWAS on an EHR-derived depression cohort using data from the eMERGE network (Auer et al., 2016; Stanaway et al., 2019). The EHR-derived depression algorithm is developed and manually validated by Kaiser Permanente and the University of Washington (KPUW), and implemented across nine eMERGE network sites. By employing modern genetic and functional analysis tools, we identified and annotated novel genetic variants in the MHC-II and IGHV regions associated with depression susceptibility. Last but not least, we showed that the EHR-derived depression cohort exhibited a strong genetic correlation with the existing large meta-analysis cohort (MDD 2019, rg = 0.8317, p = 1.7758e-11), further validating the performance of the phenotyping algorithm.

## Results

### An EHR-based depression phenotyping algorithm

The EHR-based depression algorithm was designed and developed by Kaiser Permanente in collaboration with the University of Washington (UW) eMERGE site (Supplementary Figure S1). We implemented and evaluated the performance of this algorithm internally across the eMERGE network. This algorithm achieved 100% positive predictive value (PPV) for cases and negative predictive value (NPV) for controls, including 1,999 cases and 4,268 controls. Detailed documentation of this algorithm is available from the Phenotype Knowledge website (PheKB, Supplementary Figure S1; Supplementary Table S1, and Methods) (PheKB, 2018; Kirby et al., 2016). The algorithm utilized a combination of ICD-10 and ICD-9 diagnosis codes, CPT procedure codes, medications, and stringent time-sensitive rules (the 2/30/180 rules, Methods) to define patients into cases and controls (Depression, 2018b). The algorithm yields three mutually exclusive classes of cases (major depression with psychosis, major depressive disorder, and non-major depressive disorder). In the following analysis, we combined the three classes of cases and defined them as general depression cases to boost the sample size, with patients classified as controls to form the analytic cohort. In total, we included 51,163 individuals with phenotype data and matched genomic data in the eMERGE network. Detailed demographics of this cohort are included in Table 1 (European demographics in Table 2).

**TABLE 1:** Combined ancestry depression cohort demographics breakdown (n = 51,163).

N	Case	Control	Overall	P-values
	11,532	39,631	51,163	​
Site	​	​	​	P < 3e-308
Geisinger	6.0% (696)	3.8% (1,490)	4.3% (2,186)	​
Northwestern	6.2% (712)	8.3% (3,308)	7.9% (4,020)	​
Kaiser Permanente/UW	6.9% (790)	4.4% (1744)	5.0% (2,534)	​
Mayo clinic	9.5% (1,090)	14.3% (5,666)	13.2% (6,756)	​
Columbia	1.2% (144)	4.4% (1755)	3.7% (1899)	​
Vanderbilt	20.7% (2,384)	26.6% (10,561)	25.3% (12,945)	​
Marshfield	9.3% (1,071)	4.2% (1,648)	5.3% (2,719)	​
Harvard	40.3% (4,645)	34.0% (13,459)	35.4% (18,104)	​
Sex (female)	67.0% (7,728)	49.9% (19,788)	53.8% (27,516)	P < 9.67e-236
Median BMI (kg/m^2^)	23.8, 27.3, 32.9	23.1, 26.2, 29.6	23.3, 26.2, 29.9	P = 5.84e-174
Median age	59.0, 71.0, 83.0	59.0, 74.0, 84.0	59.0, 73.0, 84.0	P = 0.006
Self-reported ethnicity	​	​	​	P < 3e-308
Hispanic or latino	4.7% (538)	2.4% (946)	2.9% (1,484)	​
Not hispanic or latino	90.8% (10,475)	87.0% (34,477)	87.9% (44,952)	​
Unknown	4.5% (519)	11.0% (4,208)	9.2% (4,727)	​
Self-reported race	​	​	​	P < 3e-308
White	85.5% (9,859)	84.0% (33,284)	84.3% (43,143)	​
Black or African American	7.4% (855)	8.2% (3,246)	8.0% (4,101)	​
American indian or Alaska native	0.2% (22)	0.1% (29)	0.1% (51)	​
Not reported	<0.1% (2)	0.1% (29)	0.1% (31)	​
Asian	0.6% (78)	1.5% (607)	1.3% (685)	​
Native Hawaiian or other Pacific islander	<0.1% (1)	<0.1% (5)	<0.1% (6)	​
Unknown	6.2% (715)	6.1% (2,431)	6.1% (3,146)	​

**TABLE 2:** European cohort demographics breakdown (n = 42,515).

N	Case	Control	Overall	P-values
	9,730	32,785	42,515	
Site	​	​	​	P < 3e-308
Geisinger	1.6% (690)	3.5% (1,480)	5.1% (2,170)	​
Northwestern	1.5% (618)	6.8% (2,880)	8.2% (3,498)	​
Kaiser Permanente/UW	1.7% (710)	3.6% (1,521)	5.2% (2,231)	​
Mayo clinic	2.5% (1,056)	12.6% (5,348)	15.1% (6,404)	​
Columbia	0.1% (26)	1.1% (483)	1.2% (509)	​
Vanderbilt	4.5% (1915)	19.1% (8,128)	23.6% (10,043)	​
Marshfield	2.5% (1,052)	3.8% (1,625)	6.3% (2,677)	​
Harvard	37.6% (3,663)	34.5% (11,320)	35.2% (14,983)	​
Sex (female)	65.5% (6,374)	48.4% (15,866)	53.2% (22,240)	P = 1.92e-196
Median BMI (kg/m^2^)	23.4, 26.8, 32.4	22.5, 26.2, 28.9	22.7, 26.2, 29.7	P = 1.47e-165
Median age	60.0, 72.2, 83.0	61.0, 74.0, 84.0	61.0, 74.0, 84.0	P = 2.52e-06
Self-reported ethnicity	​	​	​	P < 3e-308
Hispanic or latino	0% (0)	0% (0)	0% (0)	​
Not hispanic or latino	96.0% (9,343)	91.6% (30,036)	92.6% (39,379)	​
Unknown	4.0% (387)	8.4% (2,749)	7.4% (3,136)	​

### Descriptive statistics of the depression cohort and GWAS result

There are 11,532 cases and 39,631 controls in the combined GWAS analysis, in total 51,163 samples. The European ancestry GWAS analysis contains 9,730 cases and 32,785 controls. There are minor differences in the age distribution between cases and controls in both the combined cohort (students’ t-test = −2.67, *P =* 7.47e-3) and the European cohort (students’ t-test = −3.43, *P =* 5.88e-4). In addition, we observed a higher median body-mass index (BMI) (students’ t-test, *P =* 9.42e-192) and a higher proportion of females in depression cases (odds = 2.04, *P* = 2.05e-234, combined ancestry; odds = 2.03, *P* = 1.92e-196, European cohort). This gender discrepancy is consistent with the well-known female predominance in the prevalence of depression (Mayo Clinic, 2019; Palanza, 2001; Albert, 2015). Researchers explored social factors and housing style (grouped versus individual) in animal models to explain the discrepancies in depression rates in gender (Palanza, 2001). Existing opposite-sex twin research excluding genetic factors identified different sensitivity levels between women and men in social factors such as interpersonal relationships and goal-oriented factors (Kendler and Gardner, 2014).

**FIGURE 1:**
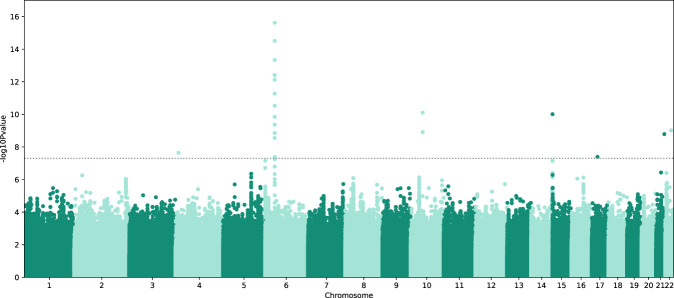
Manhattan plot of the Combined ancestry GWAS (n = 51,163).

**FIGURE 2:**
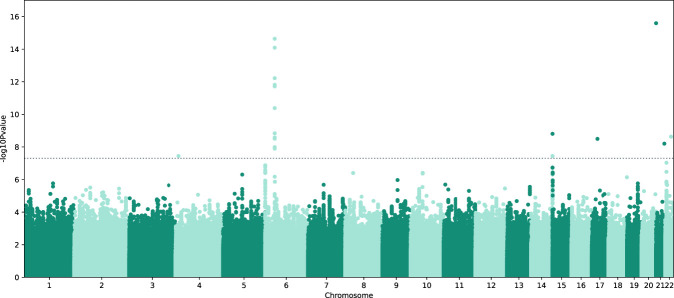
Manhattan plot of the European ancestry GWAS (n = 42,515).

The Manhattan plot of the GWAS results for the combined ancestry cohort is shown in Figure 1, with a genomic inflation factor (λ) of 1.018, and the corresponding quantile–quantile (Q–Q) plot presented in Supplementary Figure S2. We additionally report ancestry-specific GWAS results for genetically determined European ancestry (Figure 2; Supplementary Figure S3), African ancestry (Supplementary Figure S4, S5), and Asian ancestry (Supplementary Figure S6, S7). For the ancestry-stratified analyses, we included only samples with concordant self-reported and genetically determined ancestry (GDA) to ensure population consistency.

### Re-identification of genes associated with depression risk

We re-identified two genes, *PHF5A and KCNG2* (with suggestive *P <* 1e-5, Supplementary Table S1) associated with depression (in GWAS of unipolar depression, major depressive disorder, and bipolar disorder derived from the GWAS catalog) in both European and combined ancestry analysis (MacArthur et al., 2016). Although our sample size is limited compared to most large depression cohort GWAS studies, re-identifying existing genes supports the validity and practical use of the EHR-based depression phenotyping algorithm (Howard et al., 2019; Levey et al., 2021). In African ancestry GWAS (n = 3,950), we also re-identified three genes in association with depression, including *SGCZ*, *ASIC2*, and *ZC3H7A* (Supplementary Table S1), with suggestive *P* < 1e-5 (Howard et al., 2018; Fabbri et al., 2019; Heinzman et al., 2019). For Asian ancestry, limited by a small sample size (n = 583), we did not re-identify any known associations.

### Novel genetic loci for depression susceptibility

**FIGURE 3:**
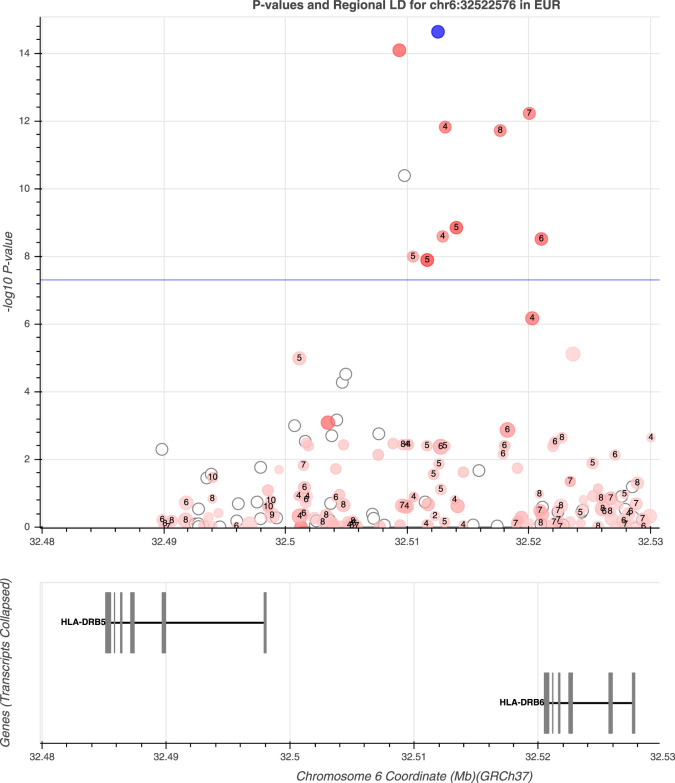
LD plot for the index SNP rs202207567 in European ancestry. The blue dot indicates the index SNP rs202207567 and other SNPs are colored in a red gradient indicating their *R* ^2^ (darker color indicates higher *R* ^2^) relative to the index SNP. The numerical values are calculated scores indicating the regulatory function of individual SNP, ranging from 0 to 10, with a higher value indicating a higher probability of having regulatory functions.

**FIGURE 4:**
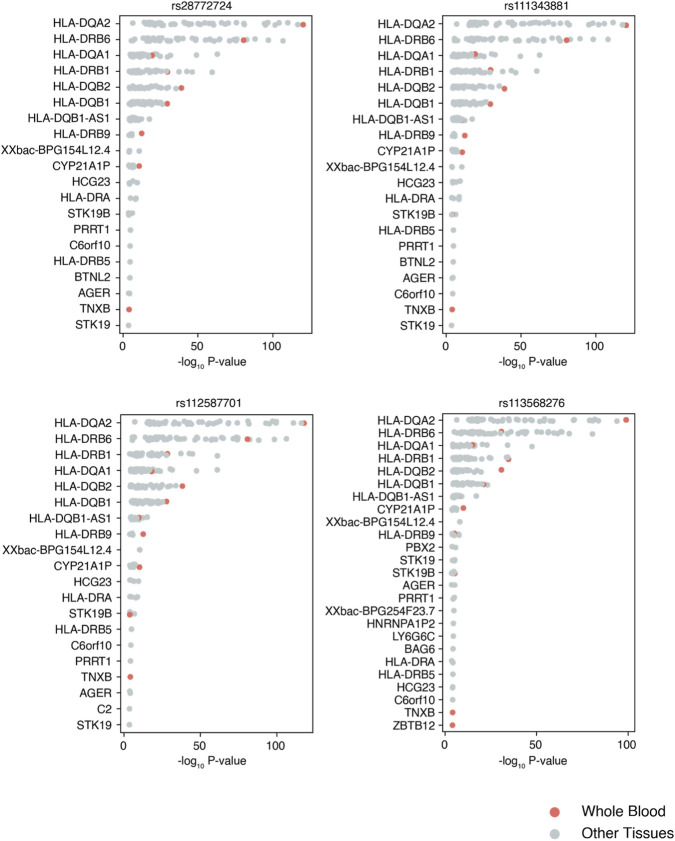
eQTLs of depression associated HLA alleles identified from GTEx. Dot plots showing eQTLs four significant SNPs within the MHC-II region. The Y-axis indicates the corresponding gene name, and the X-axis indicates the p-values of eQTLs identified in GTEX, with red representing whole-blood tissue and gray color representing other tissue types.

We identified two new loci in the combined and European ancestry analysis that had not been reported previously (Figures 1, 2). In this analysis, we focus on this strong association between the depression phenotype and several SNPs within the human leukocyte antigen (HLA) region (Figure 2) in the European cohort. In the European ancestry analysis, 18 SNPs reached genome-wide significance (*P* < 5e-8), and 11 of them (top 3 listed as rs202207567, *P* = 2.34e-15; rs28772724, *P* = 8.1e-15; rs114031016, *P* = 6.03e-15) fell in between the *HLA-DRB5* and *HLA-DRB1* genes, which belong to the MHC Class II region. Detailed summary statistics for the leading SNPs within the MHC Class II region are available in Table 3. The linkage disequilibrium (LD) plot of the index SNP (rs202207567, color in blue, Figure 3) and surrounding SNPs showed a red color gradient indicating the regulatory potential of each SNP, annotated by FORGEdb (Boyle et al., 2012). The third SNP, rs114031016, and 5th SNP, rs111365964, scored 7 and 8, respectively, indicating a high potential of having regulatory functions. Additionally, searching in the Genotype-Tissue Expression (GTEx) portal, we found multiple tissue expression quantitative trait loci (eQTL) associated with four leading SNPs (rs113568276, rs112587701, rs28772724, rs111343881). Various MHC-II class molecule eQTLs (*HLA-DQA2, HLA-DRB6, HLA-DQA1, HLA-DRB1, HLA-DQB2, HLA-DQB1*) are highly significantly associated with these 4 SNPs (Figure 4). We colored the tissue type by whole blood versus the other, as most MHC-II class molecules are expressed by antigen-presenting cells (APCs) circulating in the blood. We found that the most strongly associated eQTLs are predominantly found in Whole Blood in the GTEx portal, supporting the role of immune function in depression. The second peak within chromosome 14 had a lead SNP (rs4774137) with *P* = 3.66e-8, in the region of the *IGHV* genes. These are the immunoglobulin heavy chain variable region genes, crucial for recognizing foreign antigens and initiating immune responses. Further, we identified multiple eQTLs of this SNP (rs4774137) in the GTEx data portal. Together, we identified the HLA region and immunoglobulin heavy chain regions that are associated with depression and reached a genome-wide significance.

**TABLE 3:** Genome-Wide significant SNPs summary statistics on MHC-II region.

Chr	SNP	Ref	Alt	BP(hg19)	MAF	Logistic European P-value OR (95% CI)	Logistic combined P-value OR (95% CI)
6	rs202207567	C	T	32512533	0.13 (T)	2.33e-15 0.80 (0.68–0.93)	2.55e-16 0.81 (0.70–0.94)
6	rs28772724	G	T	32509357	0.14 (T)	8.19e-15 0.80 (0.69–0.94)	3.23e-15 0.82 (0.72–0.94)
6	rs114031016	C	T	32520035	0.13 (T)	6.03e-13 0.81 (0.70–0.95)	4.73e-14 0.83 (0.72–0.95)
6	rs113568276	G	A	32513127	0.13 (A)	1.53e-12 0.82 (0.70–0.95)	4.00e-13 0.83 (0.72–0.95)
6	rs111365964	T	G	32517646	0.13 (G)	1.93e-12 0.82 (0.70–0.95)	7.47e-13 0.83 (0.73–0.94)
6	rs76965357	T	C	32509778	0.15 (G)	4.13e-11 0.83 (0.73–0.96)	5.33e-12 0.85 (0.75–0.96)
6	rs112587701	T	A	32514041	0.16 (T)	1.43e-9 0.85 (0.74–0.97)	1.38e-10 0.86 (0.76–0.97)

### HLA association analysis

As the MHC-II region is highly polymorphic, haplotype analysis might provide a more comprehensive assessment of genetic structure. We performed association analysis on the European ancestry cohort, controlling for age, sex, median BMI, and sites, to search for HLA alleles associated with depression risk. We tested 236 HLA-alleles imputed by HIBAG (Zheng et al., 2013), including both MHC-I and MHC-II classes (*HLA-A, HLA-B, HLA-C, HLA-DPB1, HLA-DQA1, HLA-DQB1, and HLA-DRB1*). 19 alleles showed a P-value <0.05, but none were significant after Bonferroni multiple tests correction (Supplementary Table S3). Interestingly, 13 out of the 19 alleles are MHC class I molecules, including 8 *HLA-C* alleles and 5 *HLA-B* alleles, with some appearing in high frequencies (*HLA-B*:0702 23.7%, *HLA-C*:0702 25.4%). The remaining 6 alleles are MHC class II molecules, including *DQA1*, *DPB1*, and *DRB1*. Though no significant alleles passed multiple testing corrections, these results are suggestive, given that we have a relatively small sample size stratifying by each HLA molecule.

### Phenome-wide association study of tagging SNP identified various associated phenotypes and the common comorbidity of depression

**FIGURE 5:**
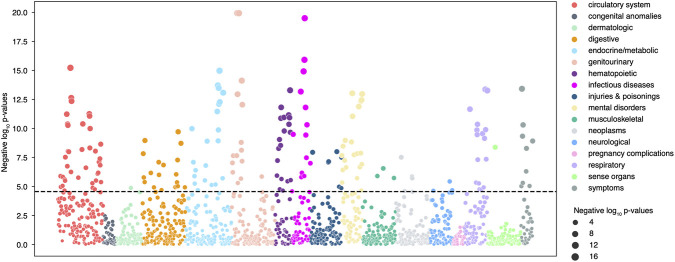
PheWAS analysis of the leading SNP is associated with over 200 traits.

We further examined the common comorbidity of depression, including several mental disorders such as bipolar, anxiety, suicidal ideation, substance abuse, tobacco disorders, schizophrenia, seizure, and PTSD. We found a significant increase in these comorbid conditions in major depression participants compared to non-major depression and controls (Supplementary Table S4). Recently, studies have also identified inflammatory phenotypes as common comorbid conditions for depression. Our analysis also revealed a higher proportion of rheumatoid arthritis, systemic lupus erythematosus, asthma, and celiac diseases in major depression compared to non-major depression and controls (Supplementary Table S5). To understand if genetics played a role in linking these conditions, we performed a Phenome-Wide Association Study (PheWAS) using the tagging SNP within the eMERGE cohort, focusing on European ancestry. The PheWAS analysis identified about 200 phenotypes significantly associated with the tagging SNP (Figure 5). We found that all the above comorbid mental disorders showed a significant association with the tagging SNP (Supplementary Table S6). However, none of the inflammatory phenotypes are significantly associated with the tagging SNP, after Bonferroni multiple-test correction (Supplementary Table S7). This result suggests that the observed genetic signal at this locus may preferentially relate to psychiatric comorbidity; however, this interpretation should be made with caution. The MHC region is highly polymorphic and characterized by extensive linkage disequilibrium, and associations observed in this region may reflect broad biological pleiotropy rather than specific, shared genetic mechanisms. Furthermore, given the polygenic architecture of depression, analyses based on a single tagging SNP are limited in their ability to capture shared genetic contributions. Therefore, while the PheWAS results provide suggestive evidence for locus-level associations with psychiatric phenotypes, they do not establish a shared genetic architecture, nor do they exclude potential genetic links between depression and inflammatory conditions.

### Genetic correlation validation of the EHR-based depression phenotyping algorithm

Finally, we performed genetic correlation analyses using LD score regression (LDSC), focusing on individuals of European ancestry to evaluate how our EHR-defined depression cohort aligns with existing large-scale meta-analyses (Bulik-Sullivan et al., 2015). Genetic correlations were estimated by comparing our cohort to three PGC European meta-analysis cohorts—MDD2019, MDD2018, and MDD 2014. The MDD2019 cohort is the largest, comprising 807,553 participants (246,363 cases and 561,190 controls) 6. MDD2018 includes 480,359 participants (135,458 cases and 344,901 controls) 4; and MDD2014 consists of 18,759 participants (9,240 cases and 9,519 controls) (Major Depressive Disorder Working Group of the Psychiatric GWAS Consortium et al., 2012). We assessed the genetic correlations between each of these PGC cohorts and our EHR-based depression phenotypes—including all depression, major depression, and non-major depression (Table 4). The most statistically significant correlation was observed between all depression phenotypes and MDD 2019 (rg = 0.8317*, P* = 1.7758e-11). Among the three reference cohorts, MDD 2019, with its larger sample size, showed the highest concordance with our data, supporting the validity and robustness of our phenotyping algorithm (Garcia, 2020).

**TABLE 4:** LDSC regression for genetic correlation analysis.

EHR derived phenotype	rg	se	z-score	p
All depression vs. MDD2018	0.7419	0.1347	5.5087	**3.6158e-08**
Major depression vs. MDD2018	0.681	0.1474	4.6206	**3.827e-06**
Non-major depression vs. MDD2018	0.6551	0.1845	3.5505	**0.0004**
All depression vs. MDD2019	0.8317	0.1237	6.7234	**1.7758e-11**
Major depression vs. MDD2019	0.8424	0.177	4.7584	**1.9512e-06**
Non-major depression vs. MDD2019	0.672	0.1469	4.5728	**4.8128e-06**
All depression vs. MDD2014	0.5612	0.2026	2.7695	**0.0056**
Major depression vs. MDD2014	0.6491	0.2402	2.7028	**0.0069**
Non-major depression vs. MDD2014	0.4326	0.2313	1.8701	0.0615

The bold values indicate significant p-values (p<0.05).

## Methods

### The EHR-based depression phenotyping algorithm

We provide an overall framework of the phenotyping algorithm, with more details documented and available on PheKB (https://phekb.org/phenotype/depression). This algorithm emphasizes the 2/30/180 rule, which can be explained as below: an individual is to have at least two diagnoses that are 30 days apart, but not over a hundred and 80 days apart. The algorithm identifies cases of depression through a partitioning workflow, starting with the most severe type--depression with psychosis (type 1). Depression with psychosis requires the individual to have a diagnosis of depression and qualifies under the 2/30/180 rule for diagnosis of depression with psychosis. If an individual fails to be eligible for type 1 depression (depression with psychosis), then we will evaluate the qualification for type 2 (major depression). Qualifying for major depression requires again, any diagnosis of depression (but failed to qualify the 2/30/180 rule for type 1) and qualifies the 2/30/180 rule for major depression diagnosis. Similarly, for type 3, non-major depression cases, individuals need to fail the previous two criteria but satisfy a 2/30/180 rule for a non-major depression diagnosis. The purpose of the 2/30/180 rule is to minimize misinterpretation of “rule-out” diagnostic codes, which might only appear once or for a brief duration (e.g., less than 30 days) and likely indicate that the diagnosis was ruled out during the diagnostic process. This approach assumes that patients with a confirmed diagnosis or prescribed a specific type of medication will exhibit recurring evidence that persists for at least 30 days. The 180-day component of the rule addresses the possibility of “rule-out” codes appearing more than once in a patient’s medical record. Isolated occurrences of depression diagnoses separated by long intervals (e.g., more than 180 days) are presumed to represent multiple rule-out episodes rather than a confirmed diagnosis of depression.

### Cohort description

We implemented the phenotyping algorithm across nine sites (Kaiser permanente/UW, Harvard University, Marshfield Clinical Research Institute, Children’s Hospital of Philadelphia, Northwestern University, Columbia University Medical Center, Major Clinic, Geisinger, and Vanderbilt University Medical Center) of the eMERGE network (including data from eMERGE Phase I to Phase III), yielding 11,532 cases and 39,631 controls in total. Detailed cohort demographics are included in Table 1.

### Genotyping and imputation

Participant samples from eMERGE-I, eMERGE-II, and eMERGE-III were genotyped and imputed. The majority of samples were genotyped with the Illumina Human 660 Quad array, while additional platforms included the CytoSNP-850K BeadChip, OmniExpress chip, Affymetrix 6.0 array, and Illumina MEGA array. The imputation is performed using the Michigan Imputation Server, which utilizes the Haplotype Reference Consortium (HRC v1.1) reference panels (Loh et al., 2016; Das et al., 2016).

### Genetically determined ancestry

Principal component analysis (PCA) was performed on all participant samples from eMERGE-I, eMERGE-II, and eMERGE-III with the PLINK 2.0 software (Chang et al., 2015). We only included common variants with >= 0.05 minor allele frequency (MAF), missingness of <= 0.1. An LD-pruned R2 threshold of 0.7 was included. The genetically determined ancestry (GDA) was defined by K-means clustering of Principal Component (PC) 1 and PC2 and three groups (corresponding to African ancestry, Asian ancestry, and European ancestry) were identified.

### GWAS and covariates

The GWAS QC steps include removing genotypes with less than 5% using--geno 0.05. HWE thresholds were set as 1e-6, with flag--hwe 1e-6 keep-fewhet. All variants with minor allele frequency (MAF) > 0.05 were kept in the analysis. In total, 9,482,808 variants were included in the analysis. The GWAS analysis included the following covariates: 1. Decade of birth (rounded as an integer); 2. Median BMI; 3. Sex; 4. Principal components (PC) 1 through 10; and 5. eMERGE site. We performed logistic regression-based association analyses for the case/control binary phenotype (general depression versus control) with the additive genotypic model of SNP genotypes coded as 0, 1, or 2 copies of the effective alleles using PLINK 2.0 (Purcell et al., 2007). The regional LD plot of the index SNP was created using the LDassoc web-based tool (Machiela and Chanock, 2018). For ancestry-specific GWAS analysis (European, African American, and Asian ancestry), we used ancestry-specific PCs as covariates and kept other covariates unchanged. All covariates were standardized using--covar-variance-standardize.

### HLA imputation and association analysis

The HLA genotype imputation is done by implementing the HIBAG 25 R package with GRCH37 references with default parameters. We obtained three MHC-I class molecules (*HLA-A, HLA-B, HLA-C*) and four MHC-II class molecules (*HLA-DRB1, HLA-DQA1, HLA-DQB1, HLA-DPB1*). We performed association analysis on the European ancestry cohort, fitting a logistic regression model for each HLA molecule predicting binary encoded depression phenotype (with 1 indicating depression and 0 indicating controls), adjusting for age, sex, median BMI, and sites to search for HLA alleles associated with depression risk.

### Phenome-wide association study on the eMERGE cohort European ancestry

We first retrieved the tagging SNP rs202207567 and encoded it by the number of alternate alleles (0, 1, or 2). Then, we mapped all diagnosis codes participants from the eMERGE cohort to phecodes and used the PheWAS package to perform a phenome-wide association study focusing only on European ancestry. The PheWAS included age, sex, median BMI, sites, and first 10 PCs as covariates.

### Common mental disorders and inflammatory comorbidity analysis

We assessed the frequency of eight mental disorders and five inflammatory phenotypes that are comorbid in depression patients. Mental disorders include bipolar (phecode = 296.1), suicidal ideation (phecode = 297), substance abuse (phecode = 316), tobacco disorder (phecode = 318), schizophrenia (phecode = 295), anxiety (phecode = 300), epilepsy (phecode = 345), and post-traumatic stress disorder (PTSD, phecode = 300.9). The inflammatory phenotypes include rheumatoid arthritis (phecode = 714), systemic lupus erythematosus (phecode = 695.42), inflammatory bowel disease (phecode = 555), asthma (phecode = 495), and celiac disease (phecode = 557.1). Supplementary Tables S5, S6 describe the raw frequency and confidence intervals for mental disorders and inflammatory phenotypes, respectively. The confidence intervals are estimated using 100 bootstrapping.

### LDSC regression analysis

LDSC regression analysis is performed to estimate the SNP-based genetic heritability (h2) and the genetic correlation between phenotypes (rg). All processes are done using munge_sumstats.py and ldsc. py, with default parameters on the European ancestry.

## Statistics

The computation of statistics is done using scipy package version 1.7.1 from Python 3.9.7. We evaluated statistical differences using a two-sided T-test (scipy.stats.ttest_ind function) for continuous variables such as age, and median BMI between depression cases and controls in both combined and European ancestry (Tables 1, 2) and Fisher’s exact test for categorical variables like sex (Tables 1, 2). We used the chi-square test (scipy.stats.chisquare) for categorical variables such as sites, race, and ethnicity.

## Discussion

In this study, we implemented an EHR-based depression phenotyping algorithm to characterize depression phenotypes within the eMERGE cohort and conducted genome-wide association analyses (GWAS). We replicated previously reported associations between genes and depression susceptibility (*PHF5A*, *KCNG2*, and others; Supplementary Table S1) and observed strong genetic concordance between our cohort and findings from large-scale meta-analyses (Table 4). These results validate the robustness of the EHR-based depression algorithm and further reveal novel genetic links between depression and immune-related pathways, thereby broadening our understanding of the biological mechanisms underlying depression.

Our findings were notable for association with loci in the MHC-II and the IGHV regions, both of which implicate key immune pathways. The MHC-II region is one of the most polymorphic regions in the human genome. The allelic diversity of the MHC-II region is crucial for the recognition of various antigens, including pathogens like bacteria, viruses, and other foreign invaders. Genes within the MHC-II region encode cell surface proteins that present antigens to T cells, thereby playing a central role in the adaptive immune response. MHC-II proteins are primarily expressed on antigen-presenting cells (APCs) such as dendritic cells, macrophages, and B cells, which capture, process, and present antigens to CD4^+^ helper T cells. The IGHV region, in turn, encodes the variable domain of the immunoglobulin heavy chain, a critical component of antibodies produced by activated B cells. Notably, both regions are integral to the adaptive immune system, suggesting a potential immunogenetic contribution to the pathophysiology of depression.

There are previously identified genetic loci in the *HLA* region and depression phenotypes (Howard et al., 2018; Thorp et al., 2021; Coleman et al., 2020). Besides, an early meta-analysis of clinical data revealed a decreased white blood cell count in depression patients (Herbert and Cohen, 1993). Thus, existing evidence points to the strong association between immune dysfunction and depression phenotypes. However, it is still unclear what the mechanisms and functional links are between depression and the immune system. One explanation for this is that the wide range of symptoms of depression might directly reflect immune dysregulation, given these links, rendering the depression phenotype as a result of mis-regulated immunity. Another aspect would focus on the psychiatric part of depression, indicating that the occurrences of depression symptoms and progressions would lead to a series of immune function dysregulation and chronic inflammation. Or it could be simply bi-directional.

Our study has a few limitations. Firstly, the study’s sample size (n = 42,515, EU) is moderate, compared to many large-scale GWAS meta-analyses of depression, limiting our power to capture previously identified loci. Also, a small sample size in African American (n = 3,950) and Asian ancestry (n = 583) made our findings less generalizable and transferable. Moreover, our phenotyping algorithm employed a stringent temporal criterion (180 days) to delineate events and encode outcomes as categorical values. This approach, while enhancing specificity, may overlook borderline cases and fail to capture the full spectrum of depression severity. A more powerful design is to incorporate the Patient Health Questionnaire-9 (PHQ-9) score and encode outcome as continuous variables reflecting the likelihood or severity of depression, thereby augmenting the detection power. Finally, the PheWAS analysis of tagging SNPs in the MHC region identified numerous associations across diverse phenotypes. However, given the highly polymorphic nature and extensive linkage disequilibrium of this region, these associations are likely to reflect biological pleiotropy and regional complexity rather than specific, interpretable links between depression and individual comorbid conditions. Accordingly, these results should be interpreted with caution.

Together, our study identified genetic variants within the MHC-II and IGHV regions that confer susceptibility to depression in individuals of European ancestry, using an EHR-based phenotyping algorithm developed by the eMERGE Consortium. We evaluated and demonstrated the robustness of this algorithm through genetic association analyses, providing values for further clinical use and depression risk prediction. Our findings suggest the role of immune regulations in depression risks, supporting prior evidence of chronic inflammation in depression and providing new insights into its underlying biological mechanisms. Future studies are needed to establish more definitive causal relationships between these genetic and immune components. Increasingly, it is evident that depression is not solely a psychiatric disorder; rather, it encompasses a complex constellation of symptoms and involves multiple physiological systems, including the immune system.

## Data Availability

The original contributions presented in the study are publicly available. This data can be found in the NCBI dbGaP repository under study accession number phs001584.v2.p2 at: https://www.ncbi.nlm.nih.gov/projects/gap/cgi-bin/study.cgi?study_id=phs001584.v2.p2.
