# Correction: GWAS analysis of a depression cohort defined by an EHR-phenotyping algorithm reveals the role of immune regulations in depression risk

**DOI:** 10.3389/fgene.2026.1916483

**Published:** 2026-07-22

**Authors:** Su Xian, David Carrell, Jordan W. Smoller, Wei-Qi Wei, Gail P. Jarvik, David R. Crosslin

**Affiliations:** 1 Department of Biomedical Informatics and Medical Education, University of Washington, Seattle, WA, United States; 2 Kaiser Permanente Washington Health Research Institute, Seattle, WA, United States; 3 Psychiatric and Neurodevelopmental Genetics Unit, Massachusetts General Hospital, Center for Genomic Medicine, Boston, MA, United States; 4 Department of Psychiatry, Massachusetts General Hospital, Center for Precision Psychiatry, Boston, MA, United States; 5 Department of Biomedical Informatics, Vanderbilt University Medical Center, Nashville, TN, United States; 6 Division of Medical Genetics, Department of Medicine, University of Washington, Seattle, WA, United States; 7 Department of Genome Sciences, University of Washington, Seattle, WA, United States; 8 Division of Biomedical Informatics and Genomics, Department of Medicine, Tulane University, New Orleans, LA, United States

**Keywords:** depression, EHR (electronic health record), genetics, GWAS, immune regulation, phenotyping algorithms

There was a mistake in Figure 5 as published. The published Figure 5 has a figure legend that covers half of one-third of the plot. The corrected Figure 5 appears below.

**FIGURE 5:**
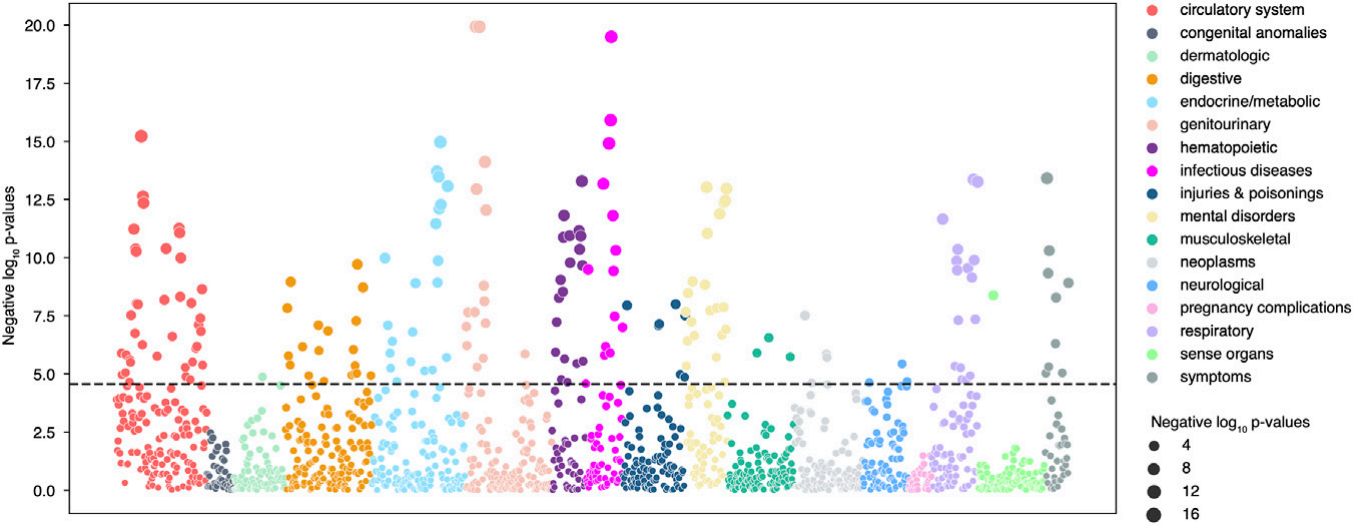
PheWAS analysis of the leading SNP is associated with over 200 traits.

There was a mistake in Table 3 as published. The published Table 3 contains duplicated information from a supplementary table, which should be removed. The corrected Table 3 appears below.

**TABLE 3:** Genome-Wide significant SNPs summary statistics on MHC-II region.

Chr	SNP	Ref	Alt	BP(hg19)	MAF	Logistic European P-value OR (95% CI)	Logistic combined P-value OR (95% CI)
6	rs202207567	C	T	32512533	0.13 (T)	2.33e-15 0.80 (0.68–0.93)	2.55e-16 0.81 (0.70–0.94)
6	rs28772724	G	T	32509357	0.14 (T)	8.19e-15 0.80 (0.69–0.94)	3.23e-15 0.82 (0.72–0.94)
6	rs114031016	C	T	32520035	0.13 (T)	6.03e-13 0.81 (0.70–0.95)	4.73e-14 0.83 (0.72–0.95)
6	rs113568276	G	A	32513127	0.13 (A)	1.53e-12 0.82 (0.70–0.95)	4.00e-13 0.83 (0.72–0.95)
6	rs111365964	T	G	32517646	0.13 (G)	1.93e-12 0.82 (0.70–0.95)	7.47e-13 0.83 (0.73–0.94)
6	rs76965357	T	C	32509778	0.15 (G)	4.13e-11 0.83 (0.73–0.96)	5.33e-12 0.85 (0.75–0.96)
6	rs112587701	T	A	32514041	0.16 (T)	1.43e-9 0.85 (0.74–0.97)	1.38e-10 0.86 (0.76–0.97)

The original version of this article has been updated.

