# Supplementary material for: GWAS analysis of a depression cohort defined by an EHR-phenotyping algorithm reveals the role of immune regulations in depression risk
Source: Front Genet. 2026 Jun 17;17:1818653. doi: 10.3389/fgene.2026.1818653 (PMC13318250; doi:10.3389/fgene.2026.1818653)
Supplement: Supplementary file 1 [file Supplementaryfile1.docx]

# Supplementary Information

### Supplementary Table 1. Diagnosis codes (ICD-9 and ICD-10) used in the depression phenotyping algorithm

| **Row** | **ICD era** | **ICD code** | **ICD description** |
| --- | --- | --- | --- |
| **Type 1. Depression diagnoses for case type 1,depression with psychosis** | | | |
| 1 | 9 | 296.34 | Major depressive affective disorder, recurrent episode, severe, specified as with psychotic behavior |
| 2 | 9 | 298.0 | Depressive type psychosis |
| 3 | 10 | F32.3 | Major depressive disorder, single episode, severe with psychotic features |
| 4 | 10 | F33.3 | Major depressive disorder, recurrent, severe with psychotic symptoms |
| **Type 2. Depression diagnoses for case type 2, major depression** | | | |
| 5 | 9 | 296.20 | Major depressive affective disorder, single episode, unspecified |
| 6 | 9 | 296.21 | Major depressive affective disorder, single episode, mild |
| 7 | 9 | 296.22 | Major depressive affective disorder, single episode, moderate |
| 8 | 9 | 296.23 | Major depressive affective disorder, single episode, severe |
| 9 | 9 | 296.25 | Major depressive affective disorder, single episode, in partial or unspecified remission |
| 10 | 9 | 296.26 | Major depressive affective disorder, single episode, in full remission |
| 11 | 9 | 296.30 | Major depressive affective disorder, recurrent episode, unspecified |
| 12 | 9 | 296.31 | Major depressive affective disorder, recurrent episode, mild |
| 13 | 9 | 296.32 | Major depressive affective disorder, recurrent episode, moderate |
| 14 | 9 | 296.33 | Major depressive affective disorder, recurrent episode, severe, without mention of psychotic behavior |
| 15 | 9 | 296.35 | Major depressive affective disorder, recurrent episode, in partial or unspecified remission |
| 16 | 9 | 296.36 | Major depressive affective disorder, recurrent episode, in full remission |
| 17 | 10 | F32.0 | Major depressive disorder, single episode, mild |
| 18 | 10 | F32.1 | Major depressive disorder, single episode, moderate |
| 19 | 10 | F32.2 | Major depressive disorder, single episode, severe without psychotic features |
| 20 | 10 | F32.4 | Major depressive disorder, single episode, in partial remission |
| 21 | 10 | F32.5 | Major depressive disorder, single episode, in full remission |
| 22 | 10 | F32.9 | Major depressive disorder, single episode, unspecified |
| 23 | 10 | F33.0 | Major depressive disorder, recurrent, mild |
| 24 | 10 | F33.1 | Major depressive disorder, recurrent, moderate |
| 25 | 10 | F33.2 | Major depressive disorder, recurrent severe without psychotic features |
| 26 | 10 | F33.40 | Major depressive disorder, recurrent, in remission, unspecified |
| 27 | 10 | F33.41 | Major depressive disorder, recurrent, in partial remission |
| 28 | 10 | F33.42 | Major depressive disorder, recurrent, in full remission |
| 29 | 10 | F33.9 | Major depressive disorder, recurrent, unspecified |
| **Type 3. Depression diagnoses for case type 3, non-major depression** | | | |
| 30 | 9 | 311 | Depressive disorder, not elsewhere classified |
| 31 | 9 | 300.4 | Dysthymic disorder |
| 32 | 9 | 309.1 | Prolonged depressive reaction |
| 33 | 10 | F32.89 | Other specified depressive episodes |
| 34 | 10 | F33.8 | Other recurrent depressive disorders |
| 35 | 10 | F43.21 | Adjustment disorder with depressed mood |
| **4. Depression diagnoses that exclude patients from qualifying as controls.** | | | |
| 36 | 9 | 296.24 | Major depressive affective disorder, single episode, severe, specified as with psychotic behavior |
| 37 | 9 | 296.82 | Atypical depressive disorder |
| 38 | 9 | 301.12 | Chronic depressive personality disorder |
| 39 | 9 | 301.13 | Cyclothymic disorder |
| 40 | 9 | 309.0 | Adjustment disorder with depression |
| 41 | 10 | F06.32 | Mood disorder due to known physiological condition with major depressive-like episode |
| 42 | 10 | F06.31 | Mood disorder due to known physiological condition with depressive features |
| 43 | 10 | F32.81 | Premenstrual dysphoric disorder |
| 44 | 10 | F34.0 | Persistent mood [affective] disorders: Cyclothymic disorder |
| 45 | 10 | F34.1 | Persistent mood [affective] disorders: Dysthymic disorder |
| 46 | 10 | F34.81 | Persistent mood [affective] disorders: Disruptive mood dysregulation disorder |
| 47 | 10 | F34.89 | Persistent mood [affective] disorders: Other specified persistent mood disorders |
| 48 | 10 | F34.9 | Persistent mood [affective] disorder, unspecified |
| 49 | 10 | F39 | Unspecified mood [affective] disorder |
| 50 | 10 | F43.23 | Adjustment disorder with mixed anxiety and depressed mood |
| 51 | 10 | F53 | Puerperal psychosis |

### Supplementary Table 2. Re-identified genes that are associated with depression from GWAS catalog

***P*<1e-5 in bold text**

| Gene | Reported traits | SNP (in this study) | P-value (Combined) | P-value (European) | P-value (African) | P-value  (Asian) |
| --- | --- | --- | --- | --- | --- | --- |
| *PHF5A* | Unipolar depression [(Yu et al., 2021)](https://paperpile.com/c/COyCuB/v2pY) | rs11705068 | ***P*=9.90e-10** | ***P*=2.28e-9** | *P*=0.057 | *P*=0.45 |
| *KCNG2* | Unipolar depression [(Howard et al., 2019; Mitchell et al., 2022)](https://paperpile.com/c/COyCuB/B1Nm+lrPV),  Major depressive disorder [(Yao et al., 2021)](https://paperpile.com/c/COyCuB/P5ox) | rs35615281 | *P=*1.30e-5 | ***P=*7.00e-7** | *P=*0.84 | *P=*0.26 |
| *ASIC2* | Unipolar depression [(Howard et al., 2018; Levey et al., 2021)](https://paperpile.com/c/COyCuB/WkAt+7Mmm) | rs16968234 | *P*=1.96e-4 | *P*=0.27 | ***P*=8.80e-7** | NA (constant allele) |
| *SGCZ* | Unipolar depression [(Fabbri et al., 2019a)](https://paperpile.com/c/COyCuB/ru0I),  Depressive symptom measure [(Fabbri et al., 2019b)](https://paperpile.com/c/COyCuB/kMHE), | rs1383411 | *P*=0.027 | *P*=0.48 | ***P*=3.11e-7** | *P*=0.43 |
| ZC3H7A | Unipolar depression [(Heinzman et al., 2019)](https://paperpile.com/c/COyCuB/ZWXe) | rs11644981 | *P=*0.01 | *P=*0.16 | ***P=*2.33e-6** | *P=*0.88 |

### Supplementary Table 3. Top hits of HLA association analysis summary statistics

| **Alleles** | **Coefficient** | **P-value** | **Fractions** | **Fractions in depression cases** |
| --- | --- | --- | --- | --- |
| DQA10601 | 0.500750 | 0.002938 | 0.010476 | 0.005341 |
| C0801 | 1.179089 | 0.005699 | 0.004193 | 0.000716 |
| C0403 | 1.933369 | 0.006234 | 0.000764 | 0.000269 |
| B1516 | 0.882931 | 0.010447 | 0.007251 | 0.001134 |
| DPB11901 | 0.292013 | 0.010545 | 0.009914 | 0.012473 |
| DRB10803 | 0.464834 | 0.011019 | 0.005089 | 0.004595 |
| C0304 | 0.085561 | 0.015342 | 0.136042 | 0.147913 |
| DQB10504 | 0.584281 | 0.015477 | 0.002066 | 0.002477 |
| B0702 | -0.069634 | 0.016198 | 0.210997 | 0.237609 |
| C0501 | -0.080481 | 0.019164 | 0.143233 | 0.168561 |
| C0702 | -0.064044 | 0.022312 | 0.230240 | 0.254230 |
| DRB11102 | 0.444519 | 0.025331 | 0.018419 | 0.003909 |
| C0303 | 0.087626 | 0.033760 | 0.091582 | 0.106675 |
| B4701 | 0.359414 | 0.038906 | 0.004205 | 0.005252 |
| B4001 | 0.081300 | 0.040594 | 0.093111 | 0.116134 |
| C1403 | 1.217580 | 0.042057 | 0.001111 | 0.000358 |
| DQA10302 | 0.131090 | 0.044974 | 0.027605 | 0.034076 |
| B3543 | 2.447207 | 0.048754 | 0.000621 | 0.000090 |
| C1203 | 0.083981 | 0.049371 | 0.084344 | 0.097335 |

### Supplementary Table 4. Common mental disorder comorbidity frequency in the EHR depression cohort

|  | Major depression | Non-major depression | control |
| --- | --- | --- | --- |
| Bipolar | 8.81 (8.49, 9.1) | 3.91 (3.67, 4.14) | 0.33 (0.29, 0.36) |
| Suicidal ideation | 8.77 (8.5, 9.13) | 3.64 (3.4, 3.86) | 0.9 (0.86, 0.95) |
| Substance abuse | 10.16 (9.83, 10.45) | 6.38 (6.03, 6.68) | 1.24 (1.19, 1.3) |
| Tobacco disorder | 18.43 (18.07, 18.86) | 14.68 (14.24, 15.08) | 8.25 (8.12, 8.4) |
| Schizophrenia | 7.04 (6.73, 7.37) | 7.13 (6.79, 7.44) | 1.34 (1.3, 1.4) |
| Anxiety | 37.09 (36.44, 37.75) | 31.69 (31.04, 32.48) | 5.72 (5.59, 5.87) |
| Seizure | 8.22 (7.93, 8.56) | 6.69 (6.35, 7.01) | 2.84 (2.75, 2.9) |
| PTSD | 6.84 (6.58, 7.14) | 2.26 (2.04, 2.47) | 0.12 (0.1, 0.14) |

### Supplementary Table 5. Inflammatory phenotypes frequency in the EHR depression cohort

|  | Major Depression | Non major depression | Control |
| --- | --- | --- | --- |
| Rheumatoid arthritis | 5.32 (5.13, 5.65) | 4.5 (4.21, 4.78) | 2.82 (2.74, 2.9) |
| Systemic Lupus Erythematosus | 1.47 (1.36, 1.58) | 1.17 (1.06, 1.32) | 0.85 (0.8, 0.89) |
| Crohn's disease | 2.58 (2.4, 2.79) | 2.22 (2.0, 2.41) | 1.59 (1.53, 1.65) |
| Asthma | 15.41 (15.08, 15.77) | 12.36 (11.89, 12.85) | 5.86 (5.75, 5.99) |
| Celiac disease | 0.72 (0.62, 0.83) | 0.41 (0.32, 0.51) | 0.34 (0.31, 0.37) |

### Supplementary Table 6. PheWAS result of the leading SNP on common mental disorders

| phecodes | phenotype | -log_10_ p-values | p-values |
| --- | --- | --- | --- |
| 296.1 | Bipolar | 4.656513 | 2.205401e-05* |
| 297 | Suicidal ideation or attempt | 6.912033 | 1.224524e-07* |
| 316 | Substance addiction and disorders | 6.639998 | 2.290876e-07* |
| 318 | Tobacco use disorder | 6.419034 | 3.810359e-07* |
| 295 | Schizophrenia and other psychotic disorders | 6.668367 | 2.146018e-07* |
| 300 | Anxiety disorders | 13.025365 | 9.432685e-14* |
| 345 | Epilepsy | 3.725833 | 1.880041e-04* |
| 300.9 | Posttraumatic stress disorder | 4.426215 | 3.747875e-05* |

*P < 0.05, Bonfferoni multiple-tests correction.

### Supplementary Table 7. PheWAS result of the tagging SNP on inflammatory phenotypes

| phecodes | phenotype | -log_10_ p-values | p-values |
| --- | --- | --- | --- |
| 714 | Rheumatoid arthritis and other inflammatory polyarthropathies | 5.898990 | 0.000001* |
| 695.42 | Systemic lupus erythematosus | 0.139674 | 0.724979 |
| 555 | Inflammatory bowel disease and other gastroenteritis and colitis | 2.673479 | 0.002121 |
| 495 | Asthma | 0.017970 | 0.959467 |
| 557.1 | Celiac disease | 1.808255 | 0.015551 |

*P < 0.05, Bonfferoni multiple-tests correction.

### Supplementary Figure 1. Flowchart of the phenotyping algorithm from [(Depression, n.d.)](https://paperpile.com/c/COyCuB/7ky6G)

###
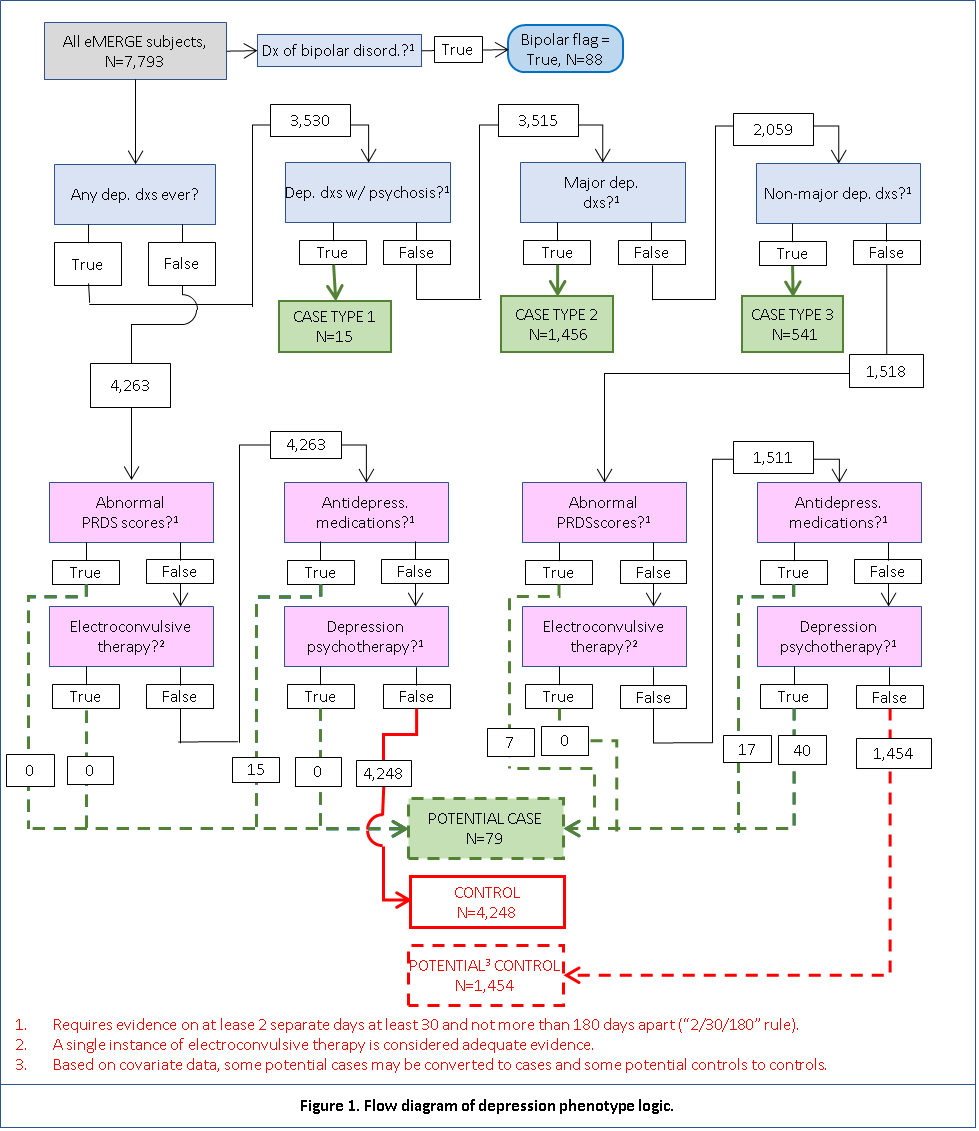


### Supplementary Figure 2 qq-plot for the combined GWAS analysis (lambda = 1.018)


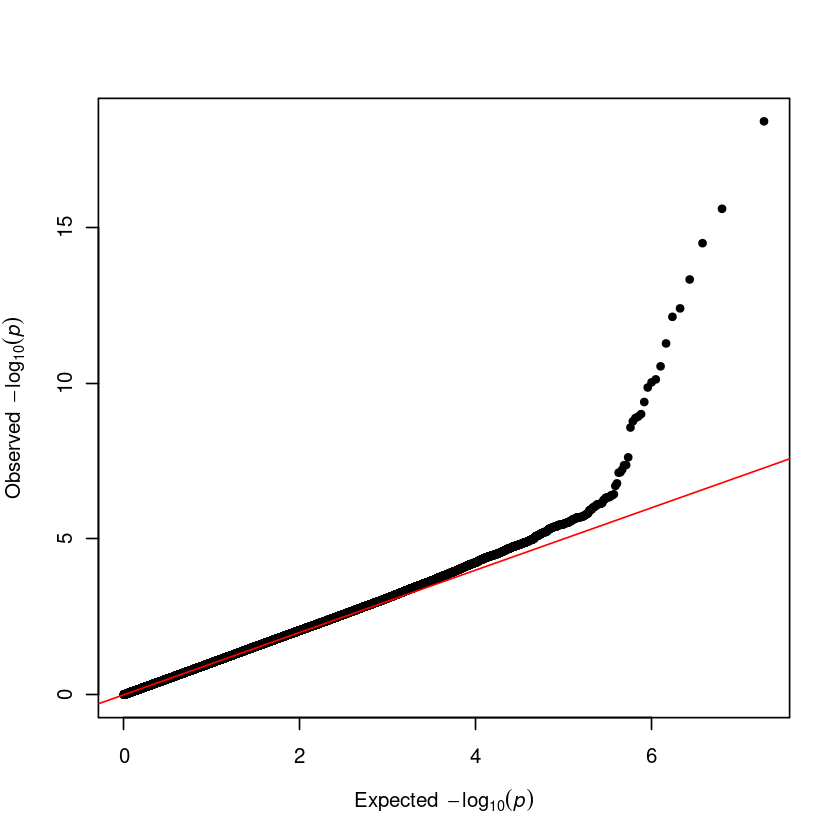


### Supplementary Figure 3 qq-plot for the European GWAS analysis


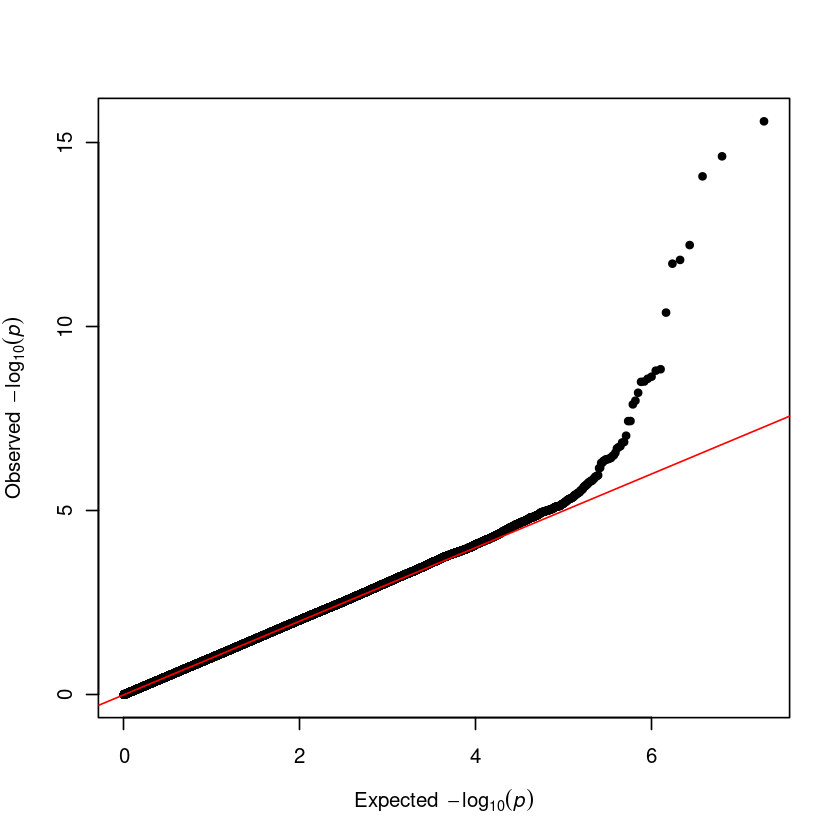


### Supplementary Figure 4. Manhattan plot of the African ancestry GWAS (n = 3950)


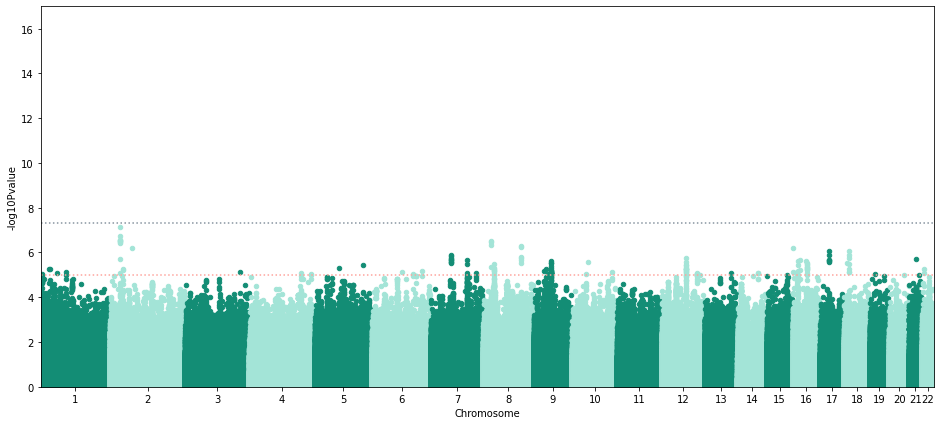


### Supplementary Figure 5. qq-plot of the African ancestry GWAS (lambda = 1.011)
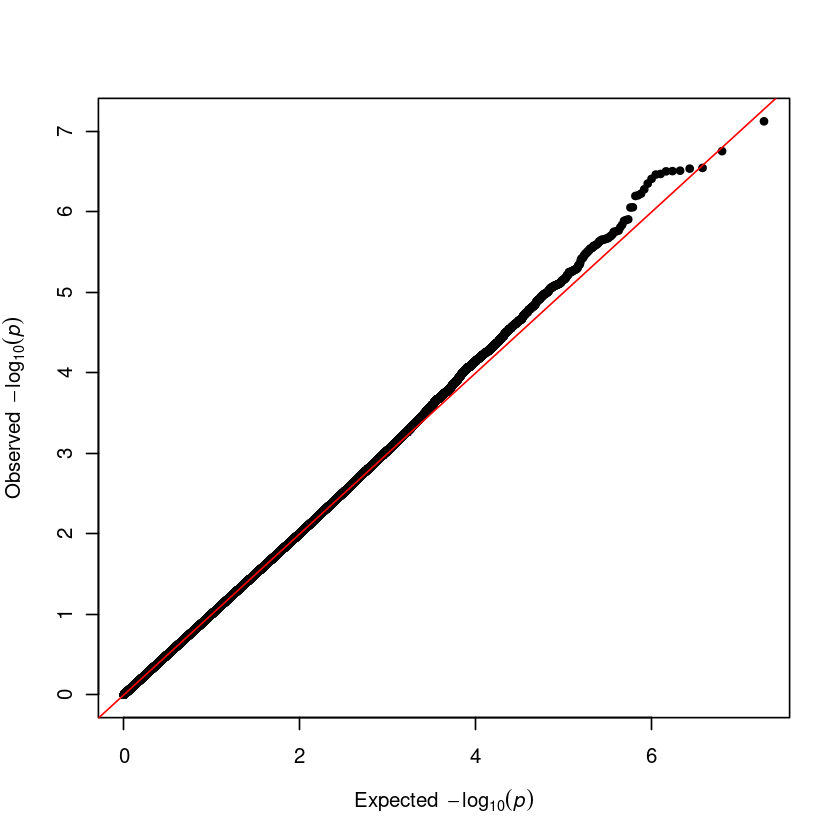


### Supplementary Figure 6. Manhattan plot of the Asian ancestry GWAS (n = 583)


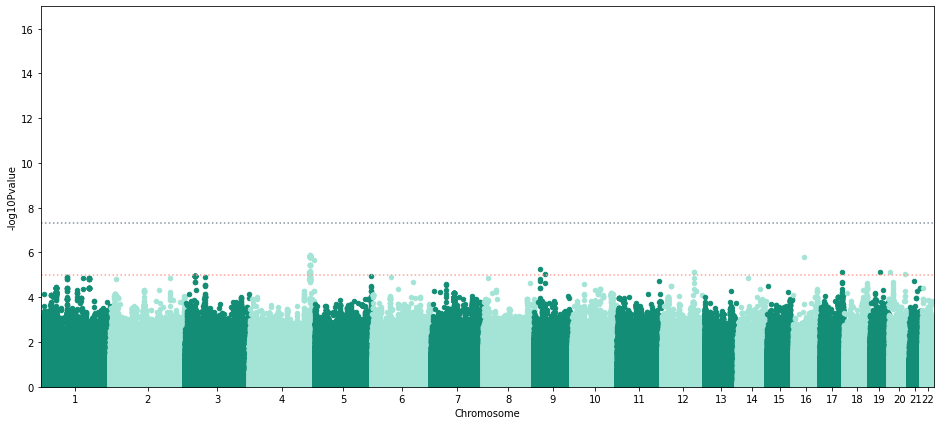


### Supplementary Figure 7. qq-plot of the Asian ancestry GWAS (lambda = 0.894)


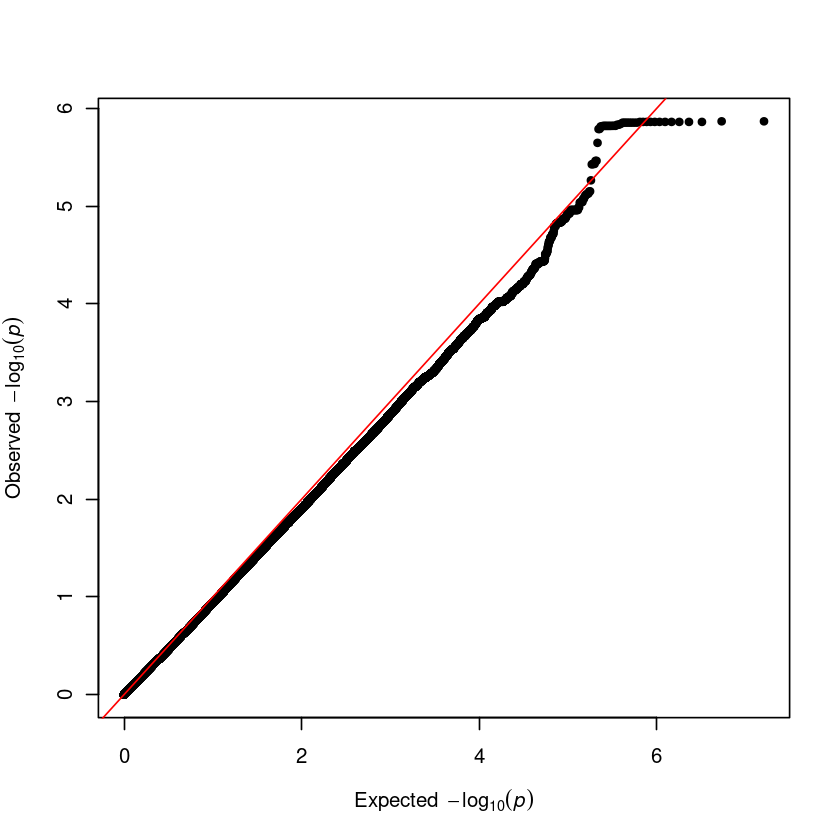


# Reference

[Depression (n.d.). Available at:](http://paperpile.com/b/COyCuB/7ky6G) <https://phekb.org/phenotype/depression> [(Accessed November 22, 2023).](http://paperpile.com/b/COyCuB/7ky6G)

[Fabbri, C., Kasper, S., Kautzky, A., Bartova, L., Dold, M., Zohar, J., et al. (2019a). Genome-wide association study of treatment-resistance in depression and meta-analysis of three independent samples. *Br. J. Psychiatry* 214, 36–41.](http://paperpile.com/b/COyCuB/ru0I)

[Fabbri, C., Kasper, S., Kautzky, A., Bartova, L., Dold, M., Zohar, J., et al. (2019b). Genome-wide association study of treatment-resistance in depression and meta-analysis of three independent samples. *Br. J. Psychiatry* 214, 36–41.](http://paperpile.com/b/COyCuB/kMHE)

[Heinzman, J. T., Hoth, K. F., Cho, M. H., Sakornsakolpat, P., Regan, E. A., Make, B. J., et al. (2019). GWAS and systems biology analysis of depressive symptoms among smokers from the COPDGene cohort. *J. Affect. Disord.* 243, 16–22.](http://paperpile.com/b/COyCuB/ZWXe)

[Howard, D. M., Adams, M. J., Clarke, T.-K., Hafferty, J. D., Gibson, J., Shirali, M., et al. (2019). Genome-wide meta-analysis of depression identifies 102 independent variants and highlights the importance of the prefrontal brain regions. *Nat. Neurosci.* 22, 343–352.](http://paperpile.com/b/COyCuB/B1Nm)

[Howard, D. M., Adams, M. J., Shirali, M., Clarke, T.-K., Marioni, R. E., Davies, G., et al. (2018). Genome-wide association study of depression phenotypes in UK Biobank identifies variants in excitatory synaptic pathways. *Nat. Commun.* 9, 1470.](http://paperpile.com/b/COyCuB/WkAt)

[Levey, D. F., Stein, M. B., Wendt, F. R., Pathak, G. A., Zhou, H., Aslan, M., et al. (2021). Bi-ancestral depression GWAS in the Million Veteran Program and meta-analysis in >1.2 million individuals highlight new therapeutic directions. *Nat. Neurosci.* 24, 954–963.](http://paperpile.com/b/COyCuB/7Mmm)

[Mitchell, B. L., Campos, A. I., Whiteman, D. C., Olsen, C. M., Gordon, S. D., Walker, A. J., et al. (2022). The Australian Genetics of Depression Study: New Risk Loci and Dissecting Heterogeneity Between Subtypes. *Biol. Psychiatry* 92, 227–235.](http://paperpile.com/b/COyCuB/lrPV)

[Yao, X., Glessner, J. T., Li, J., Qi, X., Hou, X., Zhu, C., et al. (2021). Integrative analysis of genome-wide association studies identifies novel loci associated with neuropsychiatric disorders. *Transl. Psychiatry* 11, 69.](http://paperpile.com/b/COyCuB/P5ox)

[Yu, A. Q., Wang, J., Jiang, S. T., Yuan, L. Q., Ma, H. Y., Hu, Y. M., et al. (2021). SIRT7-Induced PHF5A Decrotonylation Regulates Aging Progress Through Alternative Splicing-Mediated Downregulation of CDK2. *Frontiers in cell and developmental biology* 9. doi:](http://paperpile.com/b/COyCuB/v2pY) [10.3389/fcell.2021.710479](http://dx.doi.org/10.3389/fcell.2021.710479)
